# Nocturnal Glucose Patterns with and without Hypoglycemia in People with Type 1 Diabetes Managed with Multiple Daily Insulin Injections

**DOI:** 10.3390/jpm13101454

**Published:** 2023-09-29

**Authors:** Danil E. Kladov, Vladimir B. Berikov, Julia F. Semenova, Vadim V. Klimontov

**Affiliations:** 1 Laboratory of Endocrinology, Research Institute of Clinical and Experimental Lymphology—Branch of the Institute of Cytology and Genetics, Siberian Branch of Russian Academy of Sciences (RICEL—Branch of IC&G SB RAS), 630060 Novosibirsk, Russiaekmxtyjr@yandex.ru (J.F.S.); 2 Department of Mathematics and Mechanics, Novosibirsk State University, 630090 Novosibirsk, Russia; 3 Laboratory of Data Analysis, Sobolev Institute of Mathematics, Siberian Branch of Russian Academy of Sciences, 630090 Novosibirsk, Russia; 4 V. Zelman Institute of Medicine and Psychology, Novosibirsk State University, 630090 Novosibirsk, Russia

**Keywords:** type 1 diabetes, hypoglycemia, continuous glucose monitoring, clustering, prediction

## Abstract

Nocturnal hypoglycemia (NH) is a potentially dangerous and underestimated complication of insulin therapy. In this study, we aimed to determine which patterns of nocturnal glucose profiles are associated with NH in patients with type 1 diabetes (T1D) managed with multiple daily insulin injections. A dataset of continuous glucose monitoring (CGM) recordings obtained from 395 adult subjects with T1D was used for modeling. The clustering of CGM data was performed using a hierarchical clustering algorithm. Ten clusters without hypoglycemia and six clusters with NH episode(s) were identified. The differences among the clusters included initial and final glucose levels, glucose change during the night, and the presence of uptrends or downtrends. Post-midnight hyperglycemia was revealed in 5 out of 10 clusters without NH; in patterns with downtrends, initially elevated glucose prevented NH episodes. In clusters with initially near-normal glucose levels and downtrends, most episodes of NH were observed from midnight to 4 a.m.; if glucose was initially elevated, the episodes occurred at 2–4 a.m. or 4–6 a.m., depending on the time of the start of the downtrend. The results demonstrate the diversity of nocturnal glucose profiles in patients with T1D, which highlights the need for a differentiated approach to therapy adjustment.

## 1. Introduction

According to the International Diabetes Federation Atlas Report from 2022 [1], there are 8.75 million people living with type 1 diabetes (T1D) globally, and there were an estimated 182,000 deaths due to T1D in 2022. In real-world clinical practice, glycemic control remains sub-optimal for most people with T1D [2]. Hypoglycemia is a known barrier to achieving glycemic control goals in people with diabetes. Fear of hypoglycemia is common in subjects with T1D and affects their psychosocial well-being and diabetes management [3]. 

In the population of people with diabetes, individuals with T1D on basal–bolus insulin therapy have the greatest risk of hypoglycemia. In a recent international study evaluating the incidence of hypoglycemia in patients with insulin-treated diabetes, hypoglycemic events were reported by 97.4% of participants with T1D, with an estimated rate of 6.86 events per patient per month [4]. A growing body of evidence indicates that hypoglycemia induces a wide range of molecular and physiological changes in targeted organs, including the cardiovascular and nervous systems [5]. In patients with T1D, severe hypoglycemia is associated with a 4–10% increase in mortality [6]. Nocturnal hypoglycemia (NH) is considered to be a particular challenge, given the difficulty in recognition, prevention, and treatment. In healthy subjects, hypoglycemia usually triggers awakening, but this effect is reduced in patients with T1D [7]. An episode of NH may be a cause of sleep disturbances and morning headache, chronic fatigue, and mood changes; it is also associated with cardiac arrhythmias, resulting in “death-in-bed syndrome” [8,9]. Therefore, the prevention of NH is an important task in T1D management.

Continuous glucose monitoring (CGM) has provided new opportunities for the assessment of nocturnal glucose profiles and the detection of NH. Studies with the use of CGM showed a significantly higher prevalence of asymptomatic NH in patients with T1D than previously anticipated [10,11]. The forecasting of NH events is a difficult challenge. Previous studies indicate that both low and high glycated hemoglobin A1c (HbA1c) levels are associated with NH episodes in patients with T1D [10,11,12,13]. Additionally, glucose variability and antecedent daytime hypoglycemia turned out to be reliable predictors of NH [12,13]. Therefore, the existence of various NH patterns with different relationships with mean and pre-bedtime glucose levels and nocturnal glucose trends could be speculated.

In this study, we applied cluster analysis to CGM data to identify different patterns of interstitial glucose fluctuations at night in people with T1D managed with multiple daily insulin injections (MDIs). Clustering analysis is applied in medical research for mining knowledge and classifying patients, events, laboratory data, etc. [14]. In diabetology, cluster analysis techniques were used for the identification of diabetes subtypes [15]. In some recent studies, the clustering of CGM data was applied to different clinical tasks. Kahkoska et al. identified three dysglycemia clusters in youth with T1D based on CGM parameters measuring hypoglycemia, hyperglycemia, and glucose variability [16]. Schroder et al. applied clustering algorithms for the classification of postprandial glycemic patterns in T1D subjects under closed-loop control [17]. Tao et al. recognized four glucose clusters in patients with type 2 diabetes (T2D) based on CGM and clinical data [18]. Li et al. extracted and clustered the trend components of CGM data to assess and predict the effect of treatment on T2D [19]. In the study by Varghese et al., “time in range profile”, “hypo profile”, and “hyper profile” were identified from the CGM data of patients with T2D [20]. Inayama et al. categorized the CGM data of women with gestational diabetes into three clusters depending on glucose level and variability [21].

The aim of this study was to identify patterns of nocturnal glucose profiles with and without NH episodes by clustering CGM data in patients with T1D.

## 2. Materials and Methods

### 2.1. Database

We used a dataset of CGM recordings from the database of RICEL—a branch of IC&G SB RAS—a tertiary referral hospital. The recordings were obtained from 395 T1D patients, 153 men and 242 women, aged from 18 to 67 years, managed with MDIs. We applied the following set of exclusion criteria: current diabetic ketoacidosis, hyperglycemic hyperosmolar state, congestive heart failure (class IV according to NYHA), end-stage renal disease, known malignant neoplasm, acute infectious disease, and pregnancy. 

CGM was performed with Medtronic Paradigm MMT-722, MiniMed Paradigm Veo (MMT-754), and Medtronic CareLink Pro™ v. 2.5.A software (Medtronic, Minneapolis, MN, USA). These systems measure the level of glucose in interstitial fluid at intervals of 5 min in the range from 2.2 to 22 mmol/L.

### 2.2. CGM Data Cleaning and Preprocessing

In the first step, nocturnal (0.00–6.00) segments with a length of 72 measurements were extracted from CGM records. Time intervals with a gap of three or more consecutive values, or more than 10% of all values, were excluded. We filled missing values with an average of the nearest left and right non-zero values. If the first or last value in the time series was omitted, the gaps were filled with the nearest numeric value. 

In the next step, we divided all time series into two groups depending on the presence of NH. The NH was defined as an episode of interstitial glucose < 3.9 mmol/L lasting at least 15 min between 0 and 6 a.m. [22].

The final step of preprocessing was standardization, which consisted of transforming the glucose series from each patient into series with zero mean and unit variance.

After excluding recordings with missing data (about 17%) and outliers (4%), 2519 time series, including 256 with NH, were analyzed.

### 2.3. Clustering Procedure

By now, a number of methods for time series clustering have been proposed [23]. Taking into account the rather short length of the intervals (72 measurements for a nocturnal interval) and a large degree of glucose variability, we chose an algorithm of hierarchical clustering that allowed us to determine the structural properties of the data. The number of clusters was established empirically based on the Silhouette Score (SS) [24], visualization of solutions, and expert evaluation. 

Ward’s method was chosen as the criterion responsible for the merging classes [25]. Ward’s minimum variance criterion minimizes the total within-cluster variance. To implement this method, at each step of the clustering algorithm, we found the pair of clusters that leads to the minimum increase in total within-cluster variance after merging. This increase is a weighted squared distance between cluster centers. The Euclidean distance was chosen as the metric responsible for the distance between time series since it penalizes more for the difference in coordinate positions. Moreover, Ward’s method requires the distance between observations to be Euclidean.

### 2.4. Assessment of Clustering Quality

The evaluation of the cluster partitions was carried out by data visualization and the SS. Based on the definition of the SS, we tried to find the highest SS with a constraint on the number of clusters.

For analyzing the obtained solutions, the Monte Carlo method was applied. This is a numerical method of assessing the statistical reliability of the revealed clustering structure, which consists of testing statistical hypotheses. This method is usually applied in complicated tasks where standard techniques are impractical due to the lack of information on the statistical properties of data. We proposed a null hypothesis, which was that there is no cluster structure in our data; in other words, all observations in the dataset form one cluster. For testing the null hypothesis, we compared the quality of clustering on a real sample with the quality of clustering on synthetically generated samples of glucose data with the same number of records and lengths of time series. The quality was assessed with SS. Generation was made from a fixed probability distribution, provided that the null hypothesis is fulfilled. This meant that synthetically generated time series had no cluster structure. 

In a study by Kirilyuk IL and Senko OV [26], clustering was considered reliable for a given number of selected clusters if the index value on the real sample turned out to be greater than the value of the quantile of the *p* level for artificial data (in our case, *p* = 0.95). However, the method proposed did not take into account the fact that clustering objects are time series. Since time series usually have a dependency between neighboring components, this means that many time series have autocovariance. Thus, to assess the statistical reliability of clustering structures, we used the Monte Carlo method and took into account the autocovariance of the clustered time series for the first few lags. In our work, the autocorrelation was taken into consideration for generating time series. This made it possible to make the artificial data more similar to the real data. 

Probability distribution that was used for generating time series was multivariate Gaussian distribution N(E,C), where vector E is the sample mean, and C is a covariance matrix over the dataset. Autocorrelation was used as follows: first, over the entire time series, we found the sample means $m_{0}$, $m_{1}$, $m_{2}$, $m_{3}$ of the corresponding autocovariances at lags ${lag}_{0},\;{lag}_{1},\;{lag}_{2}$, and ${lag}_{3\;}$ (autocovariance at zero lag stands for the variance). Then, we constructed a covariance matrix as follows:

$$\left(\begin{array}{ccc} \begin{array}{cccccc} m_{0} & m_{1} & m_{2} & m_{3} & 0 & 0\\ m_{1} & m_{0} & m_{1} & m_{2} & m_{3} & 0\\ m_{2} & m_{1} & m_{0} & m_{1} & m_{2} & 0\\ m_{3} & m_{2} & m_{1} & m_{0} & m_{1} & 0\\ 0 & m_{3} & m_{2} & m_{1} & m_{0} & 0 \end{array} & \cdots & 0\\ \vdots & \ddots & \vdots \\ 0 & \cdots & \begin{array}{ccccc} m_{0} & m_{1} & m_{2} & m_{3} & 0\\ m_{1} & m_{0} & m_{1} & m_{2} & m_{3}\\ m_{2} & m_{1} & m_{0} & m_{1} & m_{2}\\ m_{3} & m_{2} & m_{1} & m_{0} & m_{1}\\ 0 & m_{3} & m_{2} & m_{1} & m_{0} \end{array} \end{array}\right)$$

Finally, we generated a multivariate Gaussian vector that is a synthetic time series. Such an approach to generation best fits our time series model since it takes into account the autocorrelation of the original time series.

### 2.5. Calculation of Quantitative Parameters of Clusters

To characterize the clusters, we calculated the initial and final glucose levels, as well as the differences between these parameters (absolute glucose change). The initial and final glucose levels were determined as the average of the first three and the last three values in each time series. For clusters with NH, the start time of an episode of hypoglycemia was also estimated. If there were two or more episodes in an interval, we recorded the time of the first event.

### 2.6. Coding

We used PyCharm for coding and Jupiter Notebook (JetBrains, Prague, Czech Republic) to visualize the solutions. The programming language was Python v. 2020.3.3. The main libraries were NumPy, pandas, SciPy, sklearn, and matplotlib.

### 2.7. Other Statistical Procedures

The Statistica 13.0 software package (Dell, Round Rock, TX, USA) was used to assess the clinical parameters of the patients. The Kolmogorov–Smirnov test was applied to test the normality. Quantitative data are presented as medians and lower and upper quartiles; frequencies are expressed as percentages (%). *p*-Values below 0.05 were considered significant.

## 3. Results

### 3.1. Clinical Characteristics of Patients 

We analyzed the results of CGM recordings from 395 patients with T1D, 153 men and 242 women, from 18 to 67 years of age (median 39 years). Diabetes duration varied from 0.5 to 55 years (median 16 years). All patients were on basal–bolus therapy with long-acting insulin analogs: glargine 100 IU/mL (n = 140), glargine 300 IU/mL (n = 135), detemir (n = 63), and degludec (n = 57). Daily insulin dose was 48 (12–132) IU/kg, or 0.7 (0.2–2.0) IU/kg. The level of HbA1c was 8.2 (7.4; 9.4)% (median (25; 75 percentile), range: 4.7–15.1%. The mean monitored glucose was 8.4 (7.3; 9.5) mmol/L; Time in Range (3.9–10 mmol/L): 68.9 (55.3; 81.7)%; Time Above Range: 30 (15.3; 42.4)%; Time Below Range: 0.6 (0.0; 1.9)%; and Coefficient of Variation 30.5 (27.2; 34.2)%.

Severe hypoglycemia in medical history was recorded in 113 (28.6%) patients. One hundred fifty individuals (38%) had impaired awareness of hypoglycemia. Other complications and diabetes-related diseases included diabetic neuropathy (n = 300, 75.9%), diabetic retinopathy (n = 218, 55.2%), chronic kidney disease (n = 216, 54.7%), peripheral artery disease (n = 72, 18.2%), arterial hypertension (n = 71, 18%), and coronary artery disease (n = 25, 6.3%). Most of the patients had a normal body mass index (BMI, 18.5–24.9 kg/m^2^), 108 were overweight (BMI 25–29.9 kg/m^2^), and 60 individuals had obesity (BMI ≥ 30 kg/m^2^).

### 3.2. Glucose Clusters without NH Episodes

**Figure 1:**
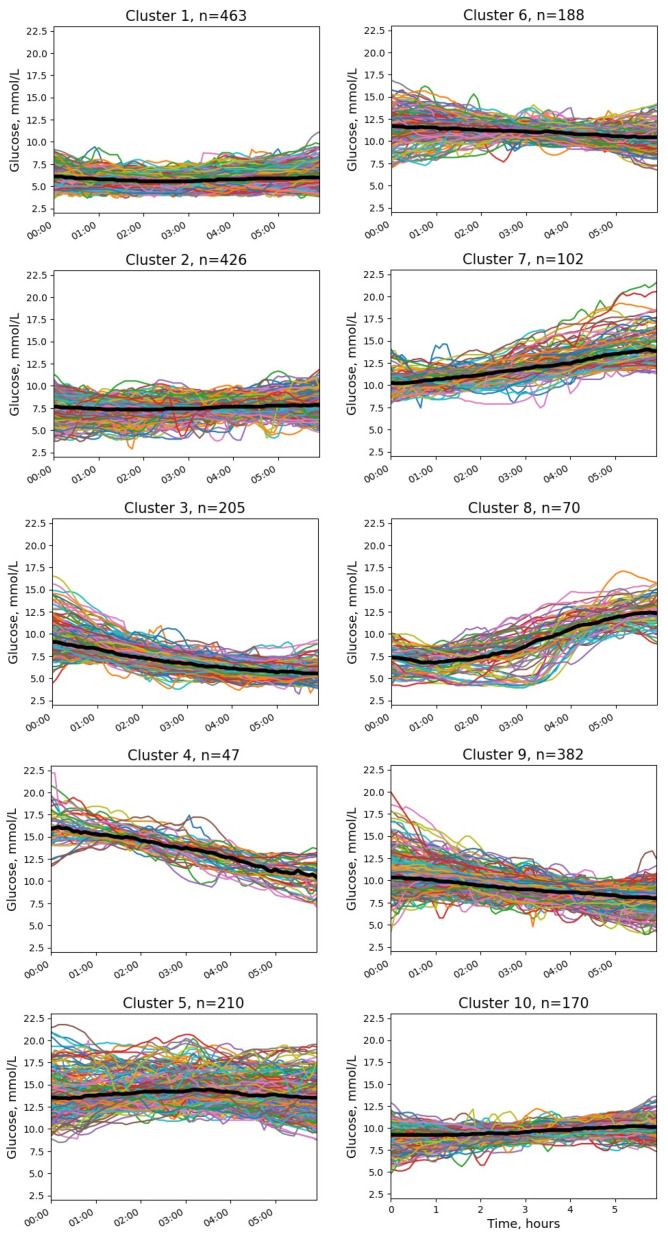
Clusters of nocturnal glucose without episodes of NH in patients with T1D.

**Figure 2:**
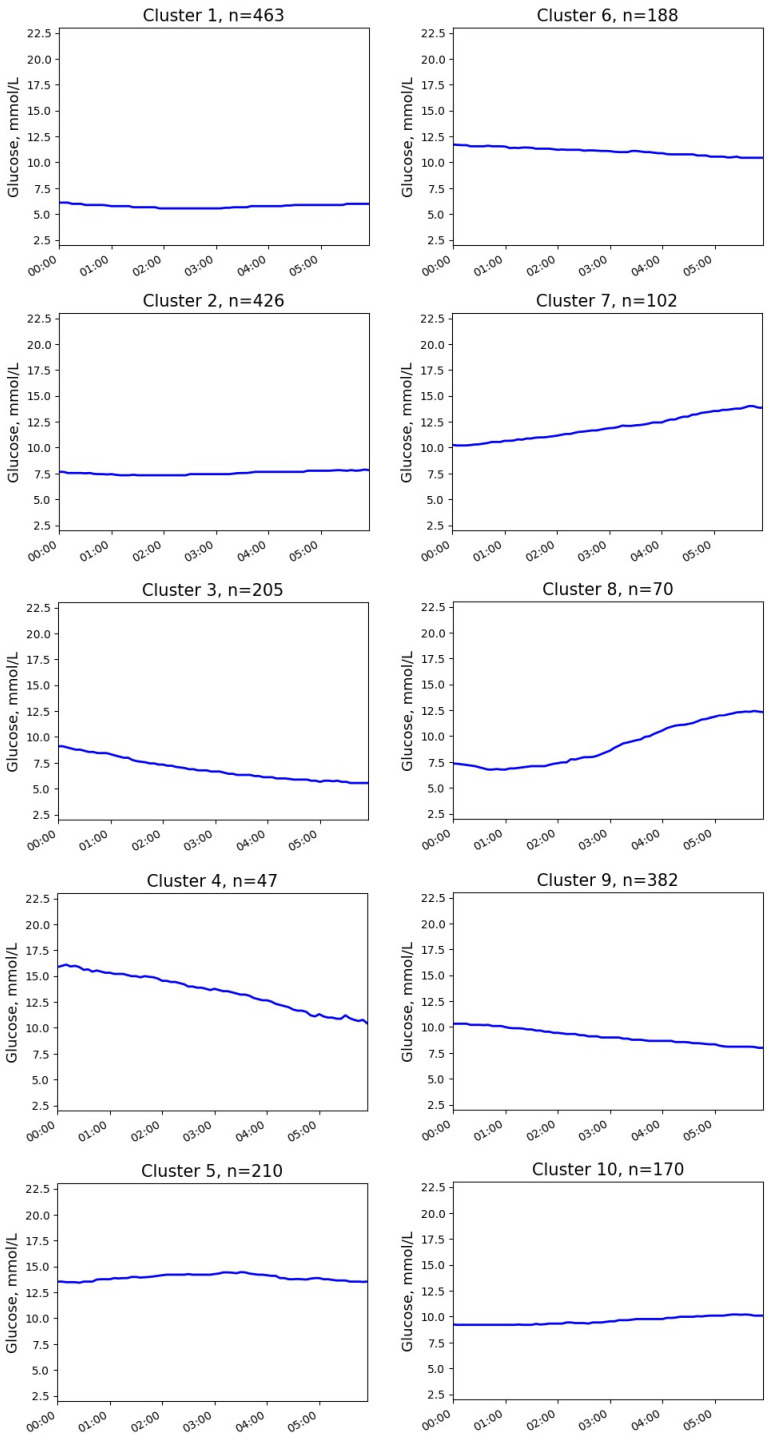
Medoids of glucose clusters without episodes of NH in patients with T1D.

Ten clusters without NH were identified (Figure 1 and Figure 2). 

The differences between the clusters included glucose levels at the start and at the end of the interval, absolute glucose changes, and the presence of ascending and/or descending glucose trends. The numerical parameters of the clusters are presented in Table 1.

The most common pattern (cluster 1 in Figure 1) demonstrated a glucose profile similar to the physiological one with normal or near-normal initial glucose and stable glucose levels during the night. Cluster 2 was also characterized by stable glucose levels, but the initial and final glucose concentrations were higher. 

Three clusters (clusters 3, 8, and 10) showed close to normal or slightly elevated glucose levels at the beginning of the time interval (mean levels < 10 mmol/L) but with different dynamics during the night. Cluster 3 was characterized by a decrease in glucose levels, cluster 8 had a pronounced upward trend, and the glucose level in cluster 10 was stable.

The next group of clusters was characterized by hyperglycemia (median >10 mmol/L) at the beginning of the time interval (clusters 4–7, 9). In cluster 7, glucose continued to rise during the analyzed interval (mean glucose change +3.4 mmol/L). On the contrary, clusters 4 and 9 showed marked downtrends (glucose change −5.6 and −2.2 mmol/L, respectively). The remaining clusters (5 and 6) represented stable hyperglycemia without significant changes in glucose levels (mean glucose change ≤1 mmol/L).

### 3.3. Glucose Clusters with NH

**Figure 3:**
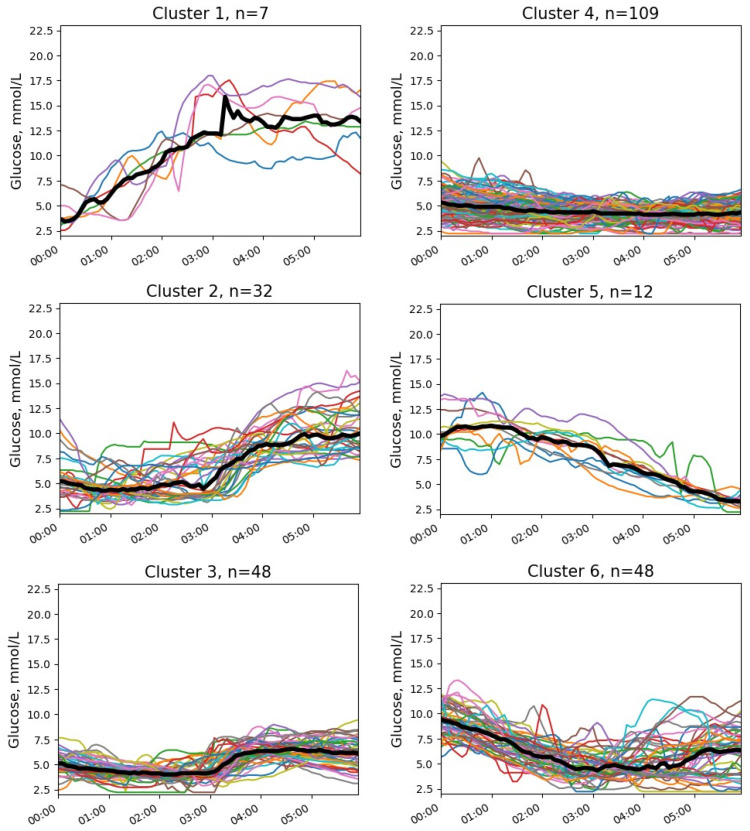
Glucose clusters with NH in patients with T1D.

**Figure 4:**
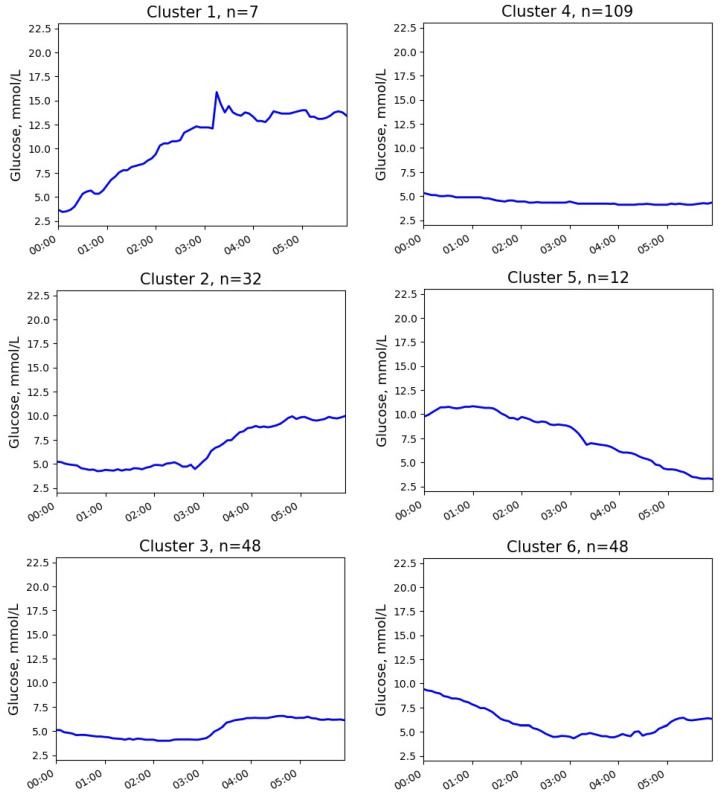
Medoids of the glucose clusters with NH in patients with T1D.

Six clusters with at least one episode of NH were identified (Figure 3 and Figure 4). The quantitative characteristics of these clusters are presented in Table 2. No episode of severe hypoglycemia was observed.

Most observations (clusters 2, 3, and 4) corresponded to the patterns with initially normal glucose levels (medians 5.2, 5.1, and 5.2 mmol/L, respectively) and gentle downtrends. In the most frequent pattern (cluster 4), episodes of NH typically happened between midnight and 3 a.m., the post-event uptrend was slight or even absent, and the glucose level at the end of the time interval was near the cut-off point of hypoglycemia (median 4.3 mmol/L). In cluster 3, NH was observed mainly in the first two hours after midnight, and the glucose level increased after the episode. In cluster 2, the episodes also typically occurred in the first two hours after midnight, followed by a marked increase in glucose levels, leading to hyperglycemia in the early morning.

Less commonly, NH episodes were revealed in recordings with post-midnight hyperglycemia (clusters 5 and 6). These clusters demonstrated the highest overnight glucose decline (median −6.8 and −2.8 mmol/L, respectively). In cluster 5, glucose levels began to decrease after 2 a.m., and episodes of hypoglycemia were observed at 4–6 a.m. On the contrary, in cluster 6, the downward trend was already present after midnight, and hypoglycemia occurred more often at 2–4 a.m.

In rare cases (cluster 1), NH was observed at the beginning of the time interval (0–1 a.m.). The initial glucose level was low (median 3.4 mmol/L). In addition, there was a clear uptrend during the night (+9.6 mmol/L).

### 3.4. Clustering Quality

**Figure 5:**
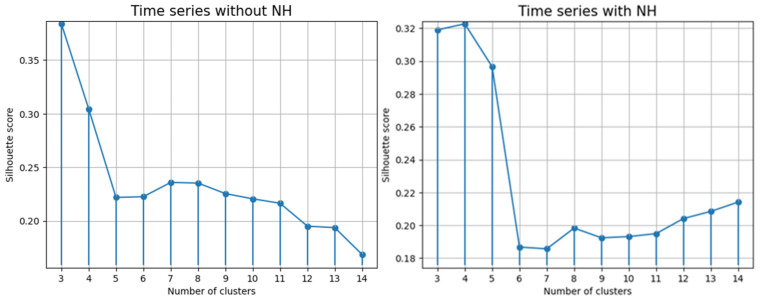
Relationships between the SS value and number of glucose clusters. NH, nocturnal hypoglycemia.

As mentioned above, the number of clusters was chosen based on the SS. The relationships between the SS and the number of glucose clusters are presented in Figure 5. 

The index value in both cases was close to 0, but nevertheless, it was a positive indication that clusters intersect and overlap each other. The time series with NH had SS values slightly higher than those without.

**Figure 6:**
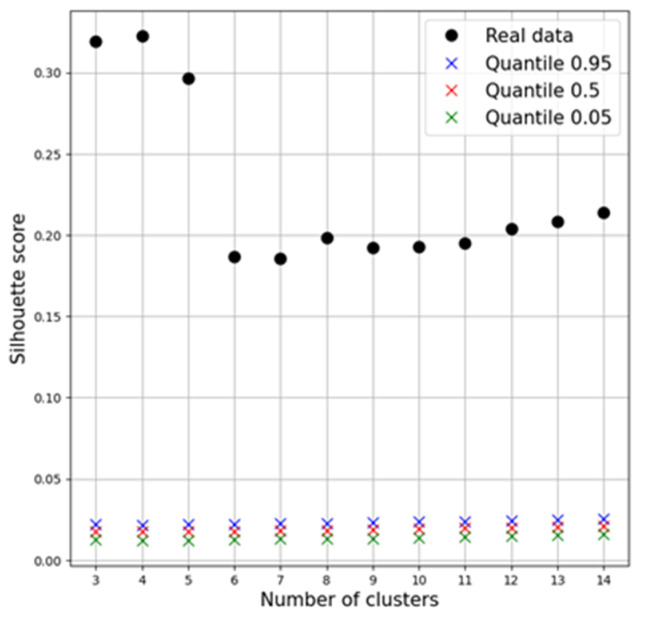
Dependence of the SS on the number of selected clusters.

The assessment of the statistical reliability of the obtained solutions showed reliable clustering at *p* < 0.05 (Figure 6).

## 4. Discussion

In this study, we performed a cluster analysis of CGM data from T1D patients managed with MDI to identify patterns of nocturnal glucose changes. We formed two groups of clusters depending on the presence of NH, a serious and hardly predictable event. We identified 10 glucose clusters without NH and 6 clusters with NH episodes. 

In previous studies, a number of algorithms were used for cluster analysis of CGM data. In particular, hierarchical clustering [21], centroid-based clustering [17,27], non-negative matrix factorization [20], and the multilevel clustering approach [18] were tested. In this study, we applied hierarchical clustering to identify different patterns of interstitial glucose fluctuations and the Monte Carlo method to assess the statistical reliability of the obtained solutions. We also took into account the autocovariance of the initial glucose time series. Thus, our study provides further development of the cluster analysis methodology as applied to CGM data.

The obtained results indicate diversity in the patterns of nocturnal glucose profiles in T1D subjects on insulin therapy. Compared to the results of other authors [18,21], we discovered more clusters in CGM data. Although the results are clinically challenging, they may provide a rationale for a new individualized approach to diabetes management with a focus on reducing the risk of NH.

In our sample, most observations of NH belonged to three clusters with normal or near-normal glucose levels after midnight (medians 5.1–5.2 mmol/L) and delicate downtrends. Most episodes of hypoglycemia with this glucose dynamics occurred from midnight to 4 a.m. Another group of clusters was characterized by hyperglycemia at the beginning of the night. In these patterns, the timing of NH episodes depended on the start time and the rate of glucose decline. Depending on the time of the start of the downtrend, the episodes occurred between 2 and 4 a.m. or 4 and 6 a.m. These data clearly demonstrate the limited value of pre-bedtime glucose as a predictor of NH. The presence of a downtrend may be a more important risk factor than pre-bedtime glucose. In agreement, the role of glucose variability as a risk factor for NH has been established [13,28]. 

The rate and amplitude of the rise in glucose levels after hypoglycemia varied significantly between clusters, affecting glucose levels in the early morning. Interestingly, in the most common glucose pattern, the episodes of NH were not accompanied by a significant increase in the post-event glucose level. Impaired glucose counter-regulation as a result of the dysfunction of regulatory mechanisms and/or excessive insulin levels may be responsible for this abnormality. It should be emphasized that normal pre-bedtime and morning glucose levels do not exclude NH episodes.

Different patterns of glucose dynamics were identified in the analysis of CGM records without NH. Most of these patterns can hardly be considered optimal due to the presence of hyperglycemia and/or downward or upward glucose trends. High glucose levels at the beginning of the night without a pronounced downtrend explain the absence of NH episodes in a number of identified clusters.

Thus, the majority of the identified nocturnal glucose patterns should be considered as motivators for revision of the treatment. Identification of the pattern of glucose fluctuations may be the basis for treatment adjustment. The presence of NH in patients with initially low glucose values or a pronounced downtrend mostly indicates an overdose of basal insulin. Hyperglycemia and/or an overnight uptrend without hypoglycemia indicate the opposite. Clusters with high pre-bedtime glucose and a pronounced downtrend are a particular problem. As a first step, the causes of high pre-bedtime glucose levels should be identified and eliminated. The persistence of a downward trend in glucose levels that is not corrected by reducing the dose of basal insulin may be an indication that a change of insulin is required. Second-generation insulin analogs (glargine 300 U/mL and degludec) provide a more stable glucose profile and may have advantages over first-generation ones in reducing the incidence of NH in patients with T1D [29,30,31]. Switching to subcutaneous continuous insulin infusion with sensor-augmented pumps or closed-loop control systems may be another option for patients with an increased risk of NH [32,33,34].

Single-site data and a relatively small sample size are the obvious limitations of our study. Nevertheless, this is the first study identifying nocturnal glucose patterns in T1D patients depending on NH. The results provide the rationale for the classification of NH depending on the time of the event, preceding glucose dynamics, and pre-bedtime and early morning glucose. Identifying a nocturnal glucose profile could be considered to be a promising approach to achieving safer and individualized diabetes care. Future research should address clinical factors that contribute to different nocturnal glucose profiles in subjects with T1D. 

## 5. Conclusions

In this study, we performed a hierarchical cluster analysis of nocturnal CGM recordings in patients with T1D depending on the presence of NH episodes. Ten clusters without NH and six clusters with NH were identified. The results clearly show a variety of nocturnal glucose patterns with and without NH and provide the rationale for the classification of NH depending on the time of the event, preceding glucose dynamics, and pre-bedtime and early morning glucose levels. Obviously, a wide range of nocturnal glucose dynamics requires a differentiated therapeutic approach. Identifying a nocturnal glucose profile could be considered a promising approach to achieving safer and individualized diabetes care.

## Figures and Tables

**Table 1:** Parameters of glucose clusters without episodes of NH in patients with T1D.

Cluster	Parameter	Median (25; 75 Percentile)	Min–Max
1	Initial glucose, mmol/L Final glucose, mmol/L Glucose change, mmol/L	6.1 (5.4; 6.8) 6.0 (5.2; 6.8) −0.2 (−1.1; 0.7)	3.8–9.0 3.9–10.8 −3.3–4.8
2	Initial glucose, mmol/L Final glucose, mmol/L Glucose change, mmol/L	7.7 (6.7; 8.4) 7.8 (7.1; 8.8) 0.3 (−0.8; 1.6)	3.8–10.9 4.9–11.5 −4.0–5.8
3	Initial glucose, mmol/L Final glucose, mmol/L Glucose change, mmol/L	9.1 (8.0; 10.4) 5.6 (5.0; 6.2) −3.4 (−4.7; −2.4)	5.0–16.4 4.0–9.2 −11.9–0.5
4	Initial glucose, mmol/L Final glucose, mmol/L Glucose change, mmol/L	15.8 (14.9; 17.4) 10.7 (9.4; 11.4) −5.6 (−7.4; −4.0)	11.9–21.0 7.5–13.7 −12.7–−1.0
5	Initial glucose, mmol/L Final glucose, mmol/L Glucose change, mmol/L	13.4 (12.2; 15.1) 13.5 (12.1; 15.1) 0.0 (−1.7; 1.6)	8.7–21.6 9.0–19.5 −7.5–7.0
6	Initial glucose, mmol/L Final glucose, mmol/L Glucose change, mmol/L	11.7 (10.4; 12.9) 10.4 (9.8; 11.1) −1.0 (−2.6; 0.4)	7.3–16.7 6.9–14.1 −8.0–5.9
7	Initial glucose, mmol/L Final glucose, mmol/L Glucose change, mmol/L	10.2 (9.5; 11.1) 14.0 (12.8; 15.3) 3.4 (2.4; 5.4)	8.2–13.7 11.3–21.2 −0.5–12.4
8	Initial glucose, mmol/L Final glucose, mmol/L Glucose change, mmol/L	7.4 (5.9; 8.4) 12.4 (11.4; 13.4) 5.4 (3.8; 6.5)	4.2–10.0 9.0–15.9 1.8–9.9
9	Initial glucose, mmol/L Final glucose, mmol/L Glucose change, mmol/L	10.3 (9.3; 11.5) 8.0 (7.3; 8.8) −2.2 (−3.7; −1.0)	5.1–19.3 4.2–13.0 −12.4–3.8
10	Initial glucose, mmol/L Final glucose, mmol/L Glucose change, mmol/L	9.2 (8.2; 10.0) 10.1 (9.5; 10.8) 1.2 (−0.1; 2.2)	5.2–12.5 7.6–13.4 −3.2–5.2

**Table 2:** Parameters of glucose clusters with NH in patients with T1D.

Cluster	Parameter	Median (25; 75 Percentile)	Min–Max
1	Initial glucose, mmol/L Final glucose, mmol/L Glucose change, mmol/L Start time of NH episode	3.4 (3.2; 4.1) 14.1 (12.7; 15.3) 9.6 (8.3; 12.4) 0 (0; 0)	2.6–7.0 8.5–16.4 5.9–12.7 0–1.1
2	Initial glucose, mmol/L Final glucose, mmol/L Glucose change, mmol/L Start time of NH episode	5.2 (4.1; 5.8) 9.8 (8.5; 12.2) 4.6 (3.3; 6.6) 0.8 (0.2; 1.6)	2.2–10.8 4.7–15.5 −2.0–11.4 0.0–3.2
3	Initial glucose, mmol/L Final glucose, mmol/L Glucose change, mmol/L Start time of NH episode	5.1 (4.6; 5.6) 6.3 (5.2; 7.2) 1.3 (0.1; 2.6) 1.3 (0.8; 2.1)	2.6–7.6 3.1–10.0 −2.7–5.3 0.0–5.7
4	Initial glucose, mmol/L Final glucose, mmol/L Glucose change, mmol/L Start time of NH episode	5.2 (4.4; 6.1) 4.3 (3.8; 4.9) −0.8 (−2.0; 0.2) 2.1 (0.6; 3.9)	2.3–9.1 2.2–7.4 −5.3–2.3 0.0–5.7
5	Initial glucose, mmol/L Final glucose, mmol/L Glucose change, mmol/L Start time of NH episode	10.0 (9.5; 10.6) 3.4 (3.2; 3.6) −6.8 (−7.4; −5.3) 5.2 (4.4; 5.5)	7.7–13.9 2.2–7.4 −5.3–2.3 0.0–5.7
6	Initial glucose, mmol/L Final glucose, mmol/L Glucose change, mmol/L Start time of NH episode	9.4 (8.2; 10.6) 6.4 (4.0; 7.9) −2.8 (−5.4; −0.4) 2.9 (2.5; 4.0)	7.7–13.9 2.2–11.1 −9.1–2.7 1.2–5.2

## Data Availability

The source data are available from the corresponding author upon request.

## Abbreviations

BMI	body mass index
CGM	continuous glucose monitoring
HbA1c	glycated hemoglobin A1c
NH	nocturnal hypoglycemia
SS	Silhouette Score
T1D	type 1 diabetes
T2D	type 2 diabetes
